# ABO Blood Type and Clinical Characteristics Among Japanese Patients With Ulcerative Colitis

**DOI:** 10.7759/cureus.59787

**Published:** 2024-05-07

**Authors:** Sen Yagi, Shinya Furukawa, Kazuhiro Tange, Tomoyuki Ninomiya, Seiyuu Suzuki, Katsuhisa Ohashi, Yasunori Yamamoto, Eiji Takeshita, Yoshio Ikeda, Yoichi Hiasa

**Affiliations:** 1 Department of Internal Medicine, Saiseikai Imabari Hospital, Imabari, JPN; 2 Health Services Center, Ehime University, Matsuyama, JPN; 3 Department of Inflammatory Bowel Diseases and Therapeutics, Ehime University, Toon, JPN; 4 Department of Gastroenterology, Ehime Prefectural Central Hospital, Matsuyama, JPN; 5 Department of Gastroenterology, Sumitomo Besshi Hospital, Niihama, JPN; 6 Surgical Gastroenterology, Ohashi Clinic, Niihama, JPN; 7 Endoscopy Center, Ehime University Hospital, Toon, JPN; 8 Department of Gastroenterology and Metabology, Ehime University Graduate School of Medicine, Toon, JPN

**Keywords:** abo blood type, japanese, inflammatory bowel disease, mucosal healing, ulcerative colitis

## Abstract

Background

The ABO blood type has been associated with several digestive diseases. Some evidence has shown an association between ABO blood type and clinical outcomes among Asian patients with Crohn’s disease. However, there are no reports about the association between ABO blood type and clinical outcomes in ulcerative colitis (UC). In this study, we aimed to evaluate the association between ABO blood type and clinical characteristics among patients with UC.

Methodology

The study subjects consisted of 277 Japanese patients with UC. Information on clinical characteristics and ABO blood type data was collected using medical records and a self-reported questionnaire. The information on clinical remission was collected using medical records. The definition of mucosal healing (MH) and partial MH was Mayo endoscopic subscore of 0 or 0-1, respectively.

Results

Of the enrolled patients, 39.4% (109/277), 18.4% (51/277), 29.2% (81/277), and 13.0% (36/277) had blood types A, B, O, and AB, respectively. The mean current age, age at onset of UC, and body mass index were 51.3 years, 42.1 years, and 22.7 kg/m^2^, and the proportion of male patients was 59.2% (164/277). The proportion of patients with clinical remission, MH, partial MH, and prednisolone use were 58.1% (161/277), 25.6% (71/277), 63.2% (175/277), and 21.3% (59/277), respectively.

Conclusions

None of the blood types were associated with any of the variables in this study. Among Japanese patients with UC, ABO blood type might not be associated with clinical characteristics.

## Introduction

Ulcerative colitis (UC) is a chronic inflammatory bowel disease (IBD). The prevalence of UC in the Asian population is increasing every year [1]. ABO antigens, which are expressed in several types of human cells and tissues [2], may interact with the pathogenesis of many disorders, including infectious, psychiatric [3], cardiovascular [4], and neoplastic diseases [5-8].

An association between the ABO blood type and digestive cancers has been reported previously. The ABO blood type is associated with the development of colorectal cancer, gastric cancer, and pancreatic cancer [5-8]. Additionally, some evidence suggests an association between ABO blood type and IBD in Asian populations. A Korean case-control study found that blood type O is a protective factor against Crohn’s disease (CD) [9]. A Chinese study found that people with blood type AB respond well to treatment for CD, while those with blood type A are more likely to fail treatment [10]. Given the association between ABO blood type and CD, we thought that ABO blood type might affect clinical outcomes among UC patients. Few studies have investigated the association between ABO blood type and UC. The frequency of ABO blood type was not associated with the onset of UC [11]. A study in Taiwan found no association between ABO blood type and clinical characteristics of UC [12]. Therefore, the present study aimed to investigate the association between ABO blood type and clinical characteristics including clinical outcomes in Japanese patients with UC.

## Materials and methods

Study population

The study subjects were 387 Japanese patients with UC from the Department of Gastroenterology and Metabology at Ehime University Graduate School of Medicine and several affiliated hospitals in Ehime Prefecture. The diagnosis of UC was based on endoscopic, radiologic, histologic, and clinical criteria. Patients with UC, whether inpatient or outpatient, who were presumed to be able to respond to the self-administered questionnaire were included. Following the exclusion of 110 patients due to incomplete data, the study’s final analysis sample comprised 277 patients, whose clinical characteristics and ABO blood type were assessed. The study protocol was approved by the institutional review board at Ehime University Graduate School of Medicine (approval number: 1505011), and written informed consent was secured from all participating patients by experienced staff. This study was also registered in the University Hospital Medical Information Network (UMIN 000051334).

Measurements

Data were collected from a self-reported questionnaire and medical records regarding endoscopic findings, drinking habits, smoking habits, onset age of UC, C-reactive protein (CRP), disease severity, clinical remission, medication, and ABO blood type. ABO blood type was divided into the following four categories: (1) type A, (2) type B, (3) type O, and (4) type AB. Blood samples, including CRP, were taken in the morning after an overnight fast. Body mass index (BMI) was calculated by dividing the weight in kilograms by the square of the height in meters.

Assessment of endoscopic activity

Mucosal status was assessed using a total colonoscopy. The Mayo Endoscopic Subscore (MES) categorizes patients into the following four levels: 0 for normal or inactive disease; 1 for mild disease with erythema, reduced vascular patterns, and mild friability; 2 for moderate disease with pronounced erythema, absent vascular patterns, friability, and erosions; and 3 for severe disease with spontaneous bleeding and ulceration. In this study, mucosal healing (MH) was defined as category 0, while partial MH was defined as categories 0 and 1. One specialist evaluated MES and MH.

Statistical analysis

Chi-square tests were employed to evaluate the associations among categorical variables. Analysis of covariance was used to compare continuous variables with categorical variables. All statistical analyses were conducted using the SAS software package version 9.4 (SAS Institute Inc., Cary, NC, USA). All statistical tests were two-tailed, and p-values <0.05 were considered statistically significant.

## Results

Table 1 shows the characteristics of the 277 study participants. The mean age of the participants was 51.3 years, and 59.2% (164/277) were male. Of the participants, 90.3% (250/277) were treated with 5-aminosalicylate, 21.3% (59/277) with prednisolone, and 5.8% (16/277) with tumor necrosis factor-alpha monoclonal antibody. Clinical remission was observed in 58.1% (161/277) of patients, MH in 25.6% (71/277), and partial MH in 63.2% (175/277).

**Table 1:** Clinical characteristics of 277 study participants. BMI = body mass index; UC = ulcerative colitis; TNF-α = tumor necrosis factor; MES = Mayo Endoscopic Subscore; MH = mucosal healing; CRP = C-reactive protein; SD = standard deviation

Variable	n (%)
Age, years, mean ± SD	51.3 ± 16.1
BMI, kg/m^2^, mean ± SD	22.7 ± 4.6
Male, n (%)	164 (59.2)
Current smoking, n (%)	21 (7.6)
Current drinking, n (%)	112 (40.4)
Age at onset of UC, years, mean ± SD	42.1 ± 16.2
Disease extent	
Pancolitis, n (%)	116 (41.9)
Left-sided, n (%)	75 (27.1)
Proctitis, n (%)	79 (28.5)
Others, n (%)	7 (2.5)
Medication	
5-aminosalicylates, n (%)	250 (90.3)
Prednisolone, n (%)	59 (21.3)
TNF-α monoclonal antibody, n (%)	16 (5.8)
Clinical remission, n (%)	161 (58.1)
MES, mean ± SD	1.18 ± 0.90
MH (MES 0), n, (%)	71 (25.6)
Partial MH (MES 0-1), n, (%)	175 (63.2)
CRP, mg/dL, mean ± SD	0.36 ± 0.81
ABO blood type	
Type A, n (%)	109 (39.4)
Type B, n (%)	51 (18.4)
Type O, n (%)	81 (29.2)
Type AB, n (%)	36 (13.0)

Table 2 shows the associations between ABO blood type and various characteristics of UC patients. The proportions of patients in clinical remission were 58.7% (64/109), 54.9% (28/51), 60.5% (49/81), and 55.6% (20/36) among patients with blood types A, B, O, and AB, respectively. The proportions of patients with MH were 28.4% (30/109), 23.5% (12/51), 24.7% (20/81), and 22.2% (8/36), respectively. These characteristics were not significantly different among patients with different blood types, indicating no association between ABO blood type and clinical characteristics.

**Table 2:** Associations between ABO blood type and characteristics of ulcerative colitis patients. *: p-value <0.05. BMI = body mass index; TNF-α = tumor necrosis factor-alpha; MES = Mayo Endoscopic Subscore; MH = mucosal healing; CRP = C-reactive protein; SD = standard deviation

Variable	Type A (n = 109)	Type B (n = 51)	Type O (n = 81)	Type AB (n = 36)	P-value
Age, years, mean ± SD	52.1 ± 15.3	49.6 ± 15.7	50.8 ± 15.3	52.1 ± 20.3	0.79
BMI, kg/m^2^, mean ± SD	22.3 ± 3.73	22.8 ± 4.33	23.4 ± 6.03	22.0 ± 3.52	0.34
Male, n, (%)	62 (56.9)	29 (56.9)	46 (56.8)	27 (75.0)	0.21
Current smoking, n (%)	7 (6.4)	4 (7.8)	5 (6.2)	5 (13.9)	0.54
Current drinking, n (%)	47 (43.1)	19 (37.3)	31 (38.3)	15 (41.7)	0.87
Onset age, years, mean ± SD	42.3 ± 16.0	40.2 ± 16.3	43.0 ± 15.4	42.2 ± 19.3	0.83
Disease extent					
Pancolitis, n (%)	45 (41.3)	22 (43.1)	33 (40.7)	16 (44.4)	
Left-sided, n (%)	28 (25.7)	12 (23.59	25 (30.9)	5 (13.9)	
Proctitis, n (%)	32 (29.4)	17 (33.3)	22 (27.2)	13 (36.1)	
Others, n (%)	4 (3.7)	0 (0.0)	1 (1.2)	2 (5.6)	0.35
Medication					
5-aminosalicylates, n (%)	99 (90.8)	45 (88.2)	73 (90.1)	33 (91.7)	0.95
Prednisolone, n (%)	25 (22.9)	13 (25.5)	13 (16.1)	8 (22.2)	0.55
TNF-α monoclonal antibody, n (%)	6 (5.5)	3 (5.9)	4 (4.9)	3 (8.3)	0.92
Clinical remission, n (%)	64 (58.7)	28 (54.9)	49 (60.5)	20 (55.6)	0.92
MES, mean ± SD	1.18 ± 0.95	1.22 ± 0.90	1.10 ± 0.80	1.33 ± 0.96	0.62
MH (MES 0), n (%)	30 (28.4)	12 (23.5)	20 (24.7)	8 (22.2)	0.84
Partial MH (MES 0-1), n (%)	68 (62.4)	32 (62.8)	55/67.9)	20 (55.6)	0.63
CRP, mg/dL, mean ± SD	0.46 ± 0.96	0.23 ± 0.46	0.28 ± 0.68	0.42 ± 0.92	0.27

## Discussion

Among Japanese patients with UC, the ABO blood type was not associated with clinical characteristics including clinical outcomes in this study.

Blood groups are determined by genetics [13]. The ABO blood type is mainly associated with digestive cancers such as colorectal cancer, gastric cancer, and pancreatic cancer [5-8]. In addition, certain blood types within this group are associated with the prognoses of specific diseases such as esophageal squamous cell carcinoma [14,15], colon cancer [16], renal cell carcinoma [17], bladder cancer [18], nasopharyngeal carcinoma [19], and pancreatic cancer [7].

The presence of the ABO blood type antigen has been reported in the fetal mucosa of the distal colon since 1988 [20], and the possibility of an association between ABO blood type and IBD was suggested even before that.

However, some evidence has shown an association between ABO blood type and IBD. In a Korean case-control study of 1,735 patients with CD and 8,074 healthy controls, the O blood type was a protective factor against CD [9]. In a Chinese study of 293 patients with CD, the proportion of patients with the ABO blood type was similar to that in the overall Chinese population [10]. Among Western populations, only one study has investigated the association between ABO blood type and CD. In an Italian and Belgian study of patients with CD, non-O blood type was associated with penetration and stricture [21].

Only two studies investigated the association between the ABO type and UC. In a UK study of 317 patients with UC, ABO blood type percentage distribution was similar to blood donors [11]. A study from Taiwan involving 129 UC patients found that there was no association between ABO blood type and UC, even though blood type A was more prevalent. Patients with blood type O had higher erythrocyte sedimentation rate levels, whereas those with blood type A had higher hemoglobin levels [12].

The association between ABO blood type and clinical outcomes of digestive diseases was found in patients with malignant tumors in several studies [5-8,14-18]. Some studies have shown that ABO blood type is associated with the onset of CD. However, no association between ABO blood type and UC has been identified yet. Thus, the findings of this study were consistent with results regarding the association between ABO blood type and UC.

The gene product of *FUT2* plays a key role in expressing the precursors of ABO antigens on the intestinal mucosa. Additionally, blood group status influences the composition of commensal microbial communities [22-24]. In Japanese and Chinese cohorts of CD patients and controls, *FUT2* polymorphism was found to be associated with CD [25,26]. In the aforementioned Italian and Belgian case-control study, however, there was no association between *FUT2* polymorphism and CD [21].

Our study has certain limitations. First, as a cross-sectional study, it is challenging to determine the chronological sequence of patients’ treatments and outcomes. Consequently, early treatment response could not be assessed. Because of the difference between the mean age and the mean age at UC diagnosis, the length of the treatment period may obscure the potential association between ABO blood type and clinical characteristics. Second, the sample size may have been too small to accurately assess the relationship between ABO blood type and clinical outcomes. Considering the poor clinical outcomes (operation) in O blood type (6.98%) and non-O blood type (4.65%) [12], and assuming a detection rate of 75% (two-tailed test, p < 0.05), the calculated sample size was 1,400. Third, the cohort had a high rate of exclusion due to missing data. Fourth, the data regarding the extraintestinal manifestations of UC is lacking. Finally, selection bias may have affected our findings. The study subjects did not accurately represent the broader population of Japanese patients with UC. The mean age, sex ratio, and drug dosage in this study were similar to those in Japanese national surveys on UC [27]. The distribution of the ABO blood type in this study was similar to that reported in a previous nationwide population study in Japan (46.0%, 21.6%, 20.6%, and 11.9% for types A, B, O, and AB, respectively) [8]. Furthermore, this cohort consisted of Japanese patients only. Thus, the present findings may not be generalizable to other populations.

## Conclusions

This study is the first report of the association between ABO blood type and UC patients in Japan. We hypothesized that ABO blood type might be associated with certain clinical characteristics in patients with UC. However, there was no difference in the characteristics of UC by blood type. Furthermore, no difference was observed in MH. ABO blood type may not be associated with clinical characteristics or treatment outcomes in Japanese patients with UC. Nevertheless, there is a need for more prospective population-based studies at the national level to better understand the blood type distribution among UC patients and its underlying physiopathological mechanisms.
